# Supplementary material for: Identification of photoperiod-induced specific miRNAs in the adrenal glands of Sunite sheep (Ovis aries)
Source: Front Vet Sci. 2022 Jul 22;9:888207. doi: 10.3389/fvets.2022.888207 (PMC9354845; doi:10.3389/fvets.2022.888207)
Supplement: Supplementary file 1 [file Data_Sheet_1.ZIP › Supplementary file/miRDeep2 file.zip!/miRDeep2 file/Know_Structure/LP42A3/oar-mir-23b.pdf]

miRBase precursor : oar-mir-23b
Total read count : 18822
oar-miR-23b read count : 18799
remaining reads : 23

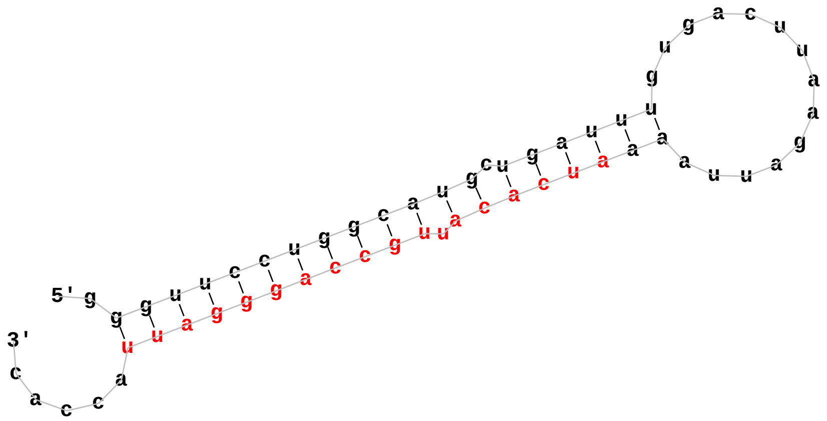

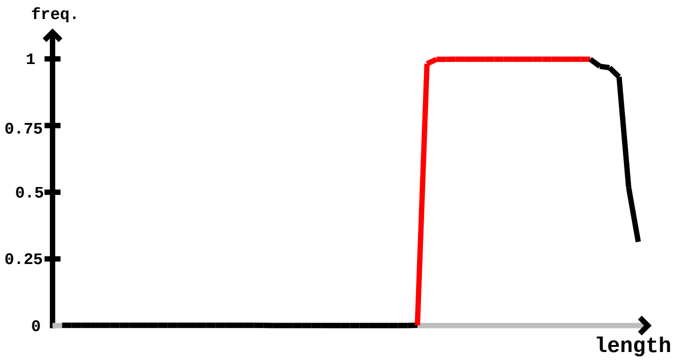


```
                                     oar-miR-23b
5'- ggguuccuggcaugcugauuugugacuuaagauuaaaaucacauugccagggauuaccac   -3'    exp
    (((((((((((((.((((((...............)))))))).))))))))))..))..    reads      mm        sample
    ggguuccuggcaugcugauu........................................    6          0         seq
    ggguAccuggcaugcugauu........................................    1          1         seq
    ggguuccuggcaugcugauuu.......................................    13         0         seq
    ....uccuggcaugcugauuugugacu.................................    1          0         seq
    ....................ugugacuuaagauuaaaa......................    1          0         seq
    .....................gugacuuaagauuaaaa......................    1          0         seq
    ...................................aaaucacauugccagggauu.....    1          0         seq
    ...................................aaaucacauugccagggauuacc..    1          0         seq
    ....................................aaucacauugccagggauu.....    4          0         seq
    ....................................aaucacauugccagggauua....    1          0         seq
    ....................................aaucacauugccagggauuac...    1          0         seq
    ....................................aaucacauugccagggauuacc..    10         0         seq
    ....................................aaucacauugccagggauuacca.    1          0         seq
    ....................................aaucacauugccagggauuaccac    4          0         seq
    .....................................aucacauugccagggau......    3          0         seq
    .....................................aucacauugcUagggauu.....    2          1         seq
    .....................................aucacauugccagggauu.....    483        0         seq
    .....................................aucacauugcUagggauua....    1          1         seq
    .....................................aucacauugccagggauua....    96         0         seq
    .....................................aucacauugccagggauuac...    636        0         seq
    .....................................aucacauugccagggauuacc..    7704       0         seq
    .....................................aucacauugccagggauuacca.    3779       0         seq
    .....................................aucacauugccagggauuaccac    5746       0         seq
    ......................................ucacauuUccagggauu.....    1          1         seq
    ......................................ucacauugccagggauu.....    6          0         seq
    ......................................ucacauugccagggauuac...    9          0         seq
    ......................................ucacauugccagggauuacc..    89         0         seq
    ......................................ucacauugccagggauuacca.    58         0         seq
    ......................................ucacauugccagggauuaccac    156        0         seq
    ........................................acauugccagggauuacca.    3          0         seq
    ........................................acauugccagggauuaccac    4          0         seq
```
